# A Quinolinone Compound Inhibiting the Oligomerization of Nucleoprotein of Influenza A Virus Prevents the Selection of Escape Mutants

**DOI:** 10.3390/v12030337

**Published:** 2020-03-19

**Authors:** Juliann Nzembi Makau, Ken Watanabe, Hiroki Otaki, Satoshi Mizuta, Takeshi Ishikawa, Yuji O. Kamatari, Noriyuki Nishida

**Affiliations:** 1 Department of Molecular Microbiology and Immunology, Graduate School of Biomedical Sciences, Nagasaki University, 1-12-4 Sakamoto, Nagasaki 852-8523, Japan; makaujuliann0@gmail.com (J.N.M.); noribaci@nagasaki-u.ac.jp (N.N.); 2 Department of Lifestyle Design, Faculty of Human Ecology, Yasuda Women’s University, 6-13-1 Yasuhigashi, Asaminami ward, Hiroshima 731-0153, Japan; 3 Center for Bioinformatics and Molecular Medicine, Graduate School of Biomedical Sciences, Nagasaki University, 1-12-4 Sakamoto, Nagasaki 852-8523, Japan; otaki@nagasaki-u.ac.jp (H.O.); s-mizuta@nagasaki-u.ac.jp (S.M.); 4 Department of Chemistry, Biotechnology, and Chemical Engineering, Graduate School of Science and Engineering, Kagoshima University, 1-21-40 Korimoto, Kagoshima 890-0065, Japan; ishi@cb.kagoshima-u.ac.jp; 5 Life Science Research Center, Gifu University, 1-1 Yanagido, Gifu 501-1193, Japan; kamatari@gifu-u.ac.jp

**Keywords:** 4-hydroxyquinolinone, antiviral, nucleoprotein, oligomerization, resistance

## Abstract

The emergence of resistance to currently available anti-influenza drugs has heightened the need for antivirals with novel mechanisms of action. The influenza A virus (IAV) nucleoprotein (NP) is highly conserved and essential for the formation of viral ribonucleoprotein (vRNP), which serves as the template for replication and transcription. Recently, using in silico screening, we identified an antiviral compound designated NUD-1 (a 4-hydroxyquinolinone derivative) as a potential inhibitor of NP. In this study, we further analyzed the interaction between NUD-1 and NP and found that the compound interferes with the oligomerization of NP, which is required for vRNP formation, leading to the suppression of viral transcription, protein synthesis, and nuclear export of NP. We further assessed the selection of resistant variants by serially passaging a clinical isolate of the 2009 H1N1 pandemic influenza virus in the presence of NUD-1 or oseltamivir. NUD-1 did not select for resistant variants after nine passages, whereas oseltamivir selected for resistant variants after five passages. Our data demonstrate that NUD-1 interferes with the oligomerization of NP and less likely induces drug-resistant variants than oseltamivir; hence, it is a potential lead compound for the development of novel anti-influenza drugs.

## 1. Introduction

Influenza A viruses (IAVs) cause seasonal infections that lead to hundreds of thousands of hospitalizations and deaths annually. Antiviral drugs are important for treatment once infection has occurred, especially in the cases of novel viruses that are not targeted by seasonal vaccines. Over the last two decades, two classes of antiviral drugs, namely, M2 ion channel and neuraminidase inhibitors, have been used globally for influenza management. However, widespread resistance to these antivirals has been reported [1,2]. Recently, new drugs targeting the RNA polymerase complex were approved for clinical use, whereas others are in clinical development [3]. In 2014, Japan approved favipiravir for limited use in complicated clinical cases [4]. Although viruses isolated from patients have not exhibited substantial reduction in favipiravir susceptibility, a recent study found that serial passage of influenza virus in the presence of favipiravir selected for resistant mutants [5]. In 2018, the cap-dependent endonuclease inhibitor baloxavir was approved in Japan and the US. A single oral dose of baloxavir is effective for treating influenza, but a point mutation (I38T) in the PA subunit that causes reduced drug sensitivity has already been reported in patients [6,7] and serial passage experiments in vitro [8]. The frequent occurrence of the I38T mutation in children younger than 12 years during treatment and the efficient human-to-human transmission of the mutant indicate the need for close monitoring regarding the clinical use of this drug [9,10,11]. Despite these advances in the discovery of influenza drugs, antivirals with novel mechanisms of action and reduced susceptibility to drug resistance are highly needed.

The IAV nucleoprotein (NP) has attracted interest as a novel antiviral target because it is a highly conserved protein playing critical roles in many stages of the viral replication cycle [12]. NP is the main component of the viral ribonucleoprotein (vRNP), a complex of viral RNA and RNA polymerase [13]. During the early stages of replication, the incoming vRNPs serve as templates for transcription and replication in the nucleus. NP is important for the nuclear import of incoming vRNPs from the cytoplasm [14]. Transcribed mRNA translocates to the cytoplasm for protein synthesis. Newly synthesized proteins (PA, PB1, PB2, and NP) are imported to the nucleus to support the assembly of progeny vRNPs. The export of progeny vRNPs to the cytoplasm for virion assembly is promoted by the interactions of NP with M1 and NS2 [15]. Through nuclear export signals, NP interacts with the cellular nuclear export receptor CRM1 to facilitate vRNP export [16]. Because of its indispensable functions during virus replication, NP represents an ideal drug target with multiple druggable binding sites.

Several small compounds have been reported to inhibit various NP functions; for example, naproxen prevents RNA binding to NP [17], a dichlorophenyl thiourea compound (compound 3) and its derivatives abrogate NP trimerization [18,19], nucleozin induces NP aggregation preventing nuclear transport of NP [20], and RK424 targets a multifunctional domain of NP, thereby inhibiting the RNA binding, trimerization, and nuclear export of NP [21]. Recently, our group explored novel NP inhibitors that bind to the NP oligomerization site via in silico screening [22]. Crystal structure studies have revealed a deep and conserved hydrophobic pocket at the site of NP oligomerization [23]. Our work led to the discovery of the 4-hydroxyquinolinone derivative NUD-1 (Figure S1A), which exhibited potent antiviral activity against IAV. In this paper, we report that NUD-1 inhibits NP oligomerization, leading to the suppression of viral transcription and protein synthesis. Moreover, NUD-1 did not select for resistant mutants following serial passage of a clinical isolate of the 2009 H1N1 pandemic influenza virus. This work identifies NUD-1 as a potential lead compound for the development of novel antiviral agents with minimal potential to select for drug-resistant mutants.

## 2. Materials and Methods

### 2.1. Cells, Viruses, and Chemicals

Madin–Darby canine kidney (MDCK) cells were maintained in minimum essential medium (Wako Pure Chemical Industries, Ltd., Tokyo, Japan) and supplemented with 5% fetal bovine serum (FBS, Life Technologies, Scoresby, Australia), 100 units/mL penicillin (Nacalai Tesque Inc, Kyoto, Japan), and 100 µg/mL streptomycin (Nacalai Tesque). Human embryo kidney 293T cells were obtained from the American Type Culture Collection (Manassas, VA, USA) and maintained in Dulbecco’s modified Eagle’s medium (DMEM, Sigma–Aldrich, St. Louis, MO, USA) containing 10% FBS. The cells were cultured at 37 °C in an atmosphere of 5% CO_2_. IAV A/WSN/33 (H1N1) was prepared as described previously [24]. The allantoic fluid of A/California/7/2009 (H1N1) was obtained from Dr. Hiroshi Kido (Tokushima University) and stored at −80 °C. Oseltamivir phosphate (F. Hoffmann-La Roche, Basel, Switzerland) and its active form oseltamivir acid (MedChem Express, Monmouth Junction, NJ, USA) were dissolved in phosphate-buffered saline (PBS). Oseltamivir refers to oseltamivir phosphate unless otherwise stated. Naproxen was purchased from Sigma–Aldrich and dissolved in ultrapure water. NUD-1 (N-(2-cyanophenyl)-1,2-dihydro-4hydroxy-2-oxo-1-pentyl-3-quinolinecarboxamide) was prepared as described previously [22] and dissolved in DMSO to a 10 mM stock. All other chemical compounds were also prepared at a stock concentration of 10 mM and stored at −30 °C until use.

### 2.2. Preparation and Purification of Recombinant NP

Recombinant NP was prepared as previously described [22]. Bacteria cell pellets were suspended and sonicated in lysis buffer containing 20 mM Tris-HCl (pH 7.9), 50 mM NaCl, 5 mM imidazole, and proteinase inhibitor cocktail (Nacalai Tesque). The lysate was treated with 0.15 mg/mL RNase A (Nacalai Tesque) at 35 °C for 30 min in the presence of 10 mM MgCl_2_. The lysate was purified using a His60 Ni Superflow Resin (Clontech Laboratories Inc, CA, USA) followed by a heparin column (Sigma–Aldrich) per the manufacturers’ instructions. The protein was eluted from the column using buffer containing 10 mM Tris-Cl (pH 7.9) and 2 M NaCl and dialyzed in storage buffer (50 mM Tris-Cl [pH 7.9] and 50 mM NaCl). The protein was stored at 4 °C for 3 days to dissociate into monomers [25], frozen in liquid nitrogen, and stored at −80 °C.

### 2.3. Analysis of the Oligomerization Status of NP

Discontinuous blue native polyacrylamide gel electrophoresis (BN-PAGE) was performed as previously described [26], with some modifications, to analyze the effect of NUD-1 on NP oligomerization in the presence of RNA. Purified NP in storage buffer and yeast tRNA (Applied Biosystems, CA, USA) in ultrapure nuclease-free water were used for the analysis. To examine the inhibition of NP oligomerization, 2.5 µM (equivalent of 2 µg) NP was mixed with varying amounts of yeast tRNA (0.15, 0.45, 1.35, and 4 µM) in the presence or absence of 100 µM NUD-1. The mixture was prepared as follows: 55.44 µL of NP was mixed with 0.56 µL of compounds and aliquoted into 4 tubes, each aliquot being 14 µL, and then 0.6 µL of RNA was added. All tested conditions contained 1% DMSO. Naproxen was included as a positive control for NP–RNA binding inhibition. The reaction mixture was kept at room temperature overnight, mixed with equal volumes of loading buffer (100 mM Tris-Cl, pH 8.0, 40% glycerol, 0.5% Coomassie brilliant blue G-250 [TCI Chemicals, Tokyo, Japan]), and incubated for 10 min. The samples were loaded onto a non-denaturing gradient gel (5%–20%) containing 200 mM Tris-Cl, pH 8.8, and run in a discontinuous buffer system (cathode buffer: 100 mM histidine [pH 8.0] and 0.002% CBB-G250; anode buffer: 100 mM Tris-Cl [pH 8.8]) at 4 °C and 15 V/cm. Gels were destained with 7.5% acetic acid and 5% ethanol. Two micrograms of proteins, thyroglobulin, apoferritin, and β-amylase (Gel filtration markers kit for protein, Sigma-Aldrich) were used as protein markers.

### 2.4. Viral Transcription Assay

The viral transcription assay was performed as previously described [27]. Briefly, 293T cells were seeded into 24-well plates at a density of 2 × 10^5^ cells/well and incubated overnight. According to the manufacturer’s instructions, cells were transfected with the following plasmids diluted using TransIT-293 (Mirus Bio LLC, Madison, WI, USA) transfection reagent: 75 ng of each of the viral protein expression plasmids (pCAGGS-PA-WSN, pCAGGS-PB1-WSN, pCAGGS-PB2-WSN, and pCAGGS-NP-WSN), 100 ng of the model viral gene expression plasmid pPolI/NP(0)GFP(0), and 1 μg of pDsRed2-monomer-N1 [28]. The medium was replaced with 500 μL of DMEM containing serially diluted compounds and 25 mM HEPES (pH 7.4) 2 h post-transfection. The next day, GFP and DsRed protein expression was observed via fluorescence microscopy (AXJ-5300TPHFL, Wraymer Inc, Osaka, Japan), and photos were taken using a USB camera (SR130, Wraymer). The numbers of GFP- and DsRed-positive cells were counted manually.

### 2.5. Indirect Immunofluorescence

Indirect immunofluorescence analysis was performed as described previously [29], with some modifications. In 24-well plates, MDCK cells (1 × 10^5^ cells/well) were seeded on coverslips and incubated overnight. At 70%–80% confluence, cells were infected with A/WSN/33 virus at a multiplicity of infection (MOI) of 5 and fixed 9 h post-infection with 4% paraformaldehyde for 10 min. Cells were permeabilized with 0.1% NP40 in PBS for 20 min followed by blocking with 1% skim milk in PBS for 40 min. Cells were reacted with anti-NP antibody (GeneTex Inc, Irvine, CA, USA), followed by Alexa Fluor 488-conjugated anti-rabbit IgG for 1 h (Invitrogen, Carlsbad, CA, USA) and observed using a fluorescence microscope.

### 2.6. Western Blotting

Analysis of viral protein expression was performed using Western blotting, as described previously [30]. Briefly, MDCK cells in 24-well plates were infected with A/WSN/33 virus (MOI = 1) in the presence or the absence of compounds. Nine hours post-infection, cells were collected, lysed, subjected to 10% SDS-PAGE, and then transferred onto a polyvinylidene fluoride membrane. The membrane was incubated with anti-HA (1:5000), anti-PA (1:5000), anti-NP (1:30,000), anti-M1 (1:30,000), or anti-actin (1:150) antibody for 4 h, followed by treatment with biotinylated secondary antibody and streptavidin alkaline phosphatase and visualization using BCIP and NBT.

### 2.7. In vitro Serial Passage of IAV

The clinical isolate of the 2009 H1N1 pandemic influenza virus strain A/California/7/2009 was serially passaged in the presence of increasing concentrations of either oseltamivir acid or NUD-1, as described [31]. In the first passage, MDCK cells were infected at a MOI of 0.001 in the presence of oseltamivir acid (0.001–0.1 µM) or NUD-1 (1–8 µM). These concentrations were selected on the basis of the 50% inhibitory concentrations (IC_50_) of oseltamivir acid (0.02 µM) and NUD-1 (2.2 µM). Cells were harvested after 3–4 days of incubation when a cytopathic effect was evident, and the viral titers were determined using the hemagglutination assay [22]. After identifying the highest concentration of oseltamivir acid or NUD-1 that permitted detectable viral growth, the supernatant from treated cells was diluted 1000-fold and used to infect new cells. The concentrations of oseltamivir acid and NUD-1 were increased by 10- and 2-fold, respectively, in subsequent passages. The 10-fold increase in the concentration of oseltamivir acid was attributable to the rapid increase in viral titers at all tested concentrations. After the fifth and ninth passages of the virus in oseltamivir acid and NUD-1, the culture supernatant was harvested. Viral titers were determined using the TCID_50_ assay [32], and the sensitivity of the passaged virus to the respective drugs was determined using crystal violet assay as described previously [33]. Isolation of a resistant clone by plaque assay in the presence of 100 µM oseltamivir was performed after the fifth passage.

### 2.8. Statistical Analysis

The results in graphs are presented as the mean ± standard deviation calculated from three independent experiments. Statistical analysis was conducted using Student’s *t*-test.

## 3. Results

### 3.1. NUD-1 Interferes with NP Oligomerization

**Figure 1:**
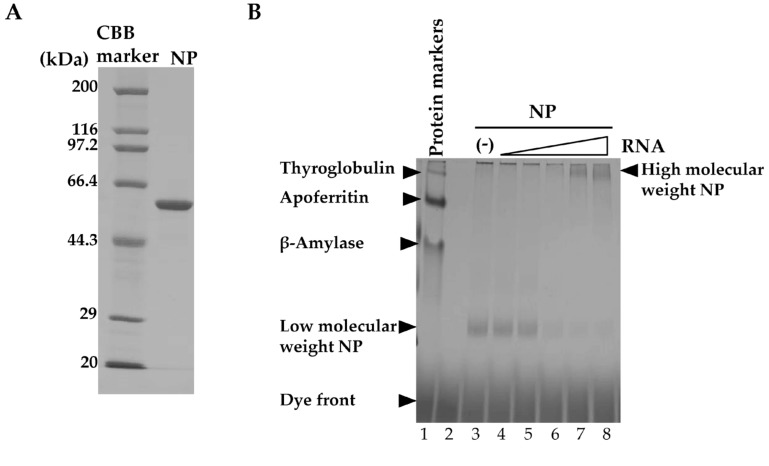

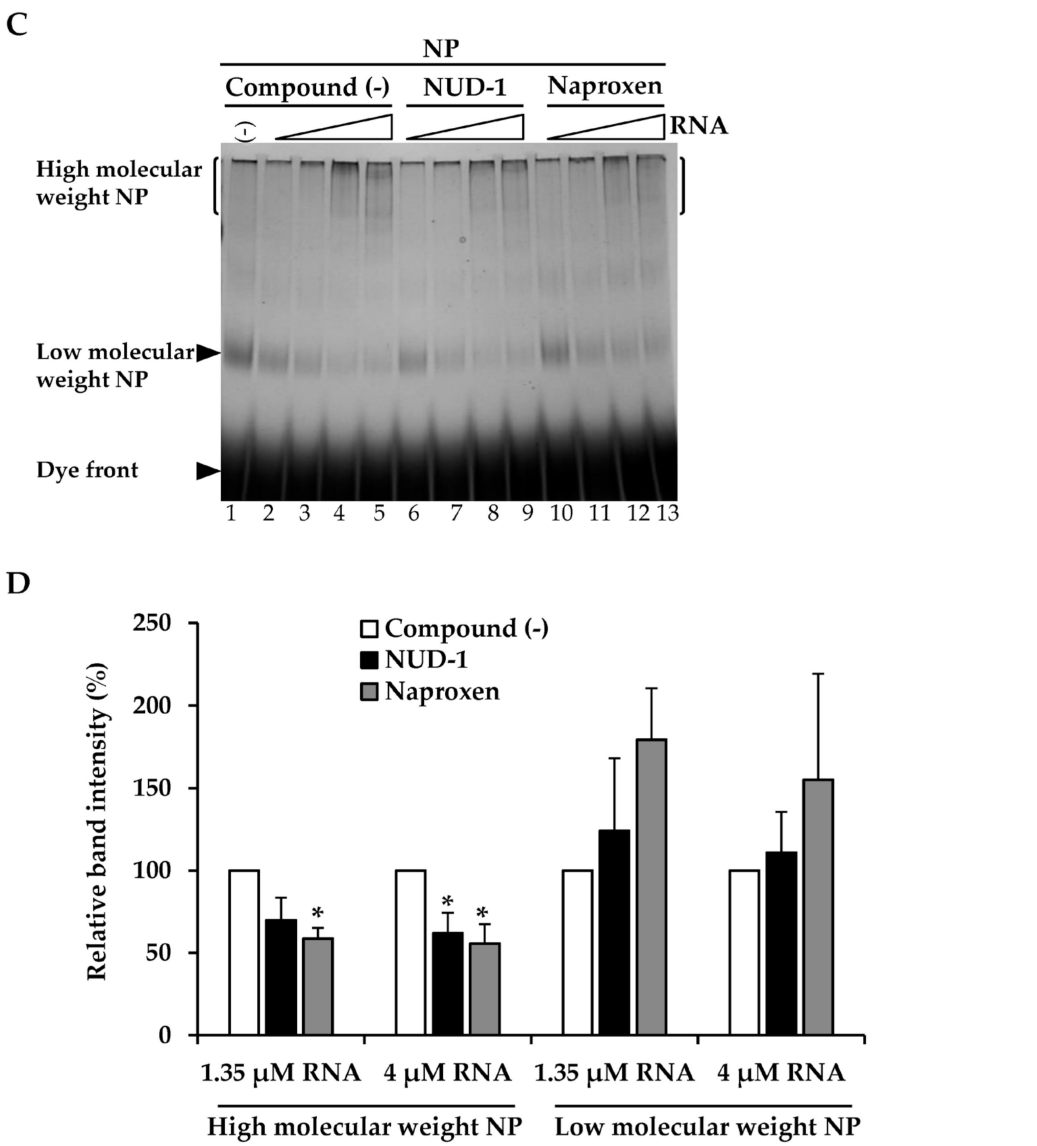
Effects of NUD-1 and naproxen on nucleoprotein (NP) oligomerization. (**A**) Purified recombinant NP was analyzed using 10% SDS-PAGE followed by Coomassie brilliant blue staining. (**B**) The migration of protein markers (thyroglobulin, 669 kDa; apoferritin, 443 kDa; β-amylase, 200 kDa) and NP mixed with yeast (0.05, 0.15, 0.45, 1.35, and 4 μM) was analyzed using blue native polyacrylamide gel electrophoresis (BN-PAGE). (**C**) NP (2.5 μM, equivalent to 2 μg) was mixed with RNA (0.15, 0.45, 1.35, and 4 μM) in the absence of any compound or in the presence of 100 μM NUD-1 or naproxen and incubated at room temperature overnight before analysis via BN-PAGE. The intensity of the smear at the top of the gel (enclosed by bracket) was quantified using ImageJ software. The relative band intensity in the presence of NUD-1 or naproxen was calculated in reference to that in the absence of a compound. Three independent experiments were performed, and representative data are shown. (**D**) The relative band intensities of high-molecular-weight and low-molecular-weight NP treated with 1.35 µM RNA (no compound, lane 4; NUD-1, lane 8; naproxen, lane 12), and NP treated with 4 µM RNA (no compound, lane 5; NUD-1, lane 9; naproxen, lane 13) were quantified from three independent experiments. The asterisk indicates *p* < 0.05.

We prepared recombinant NP and stored it in low-salt conditions to maintain it in monomeric form [25,34]. The purified NP was first analyzed by SDS-PAGE to determine its purity (Figure 1A). Only a single band corresponding to NP (56 kDa) was obtained, supporting the high purity of the NP preparation. Next, the effect of NUD-1 on NP oligomerization was investigated using BN-PAGE. NP is known to bind non-specifically to nucleic acid [23,25,34]; thus, yeast tRNA was used in this experiment. An increase in the amount of RNA (Figure 1B, lanes 4–8) resulted in a shift of the migration pattern of NP compared with that of NP in the absence of RNA (lane 3), suggesting the formation of high-molecular-weight structures [35]. The effect of NUD-1 and naproxen on RNA-induced NP oligomerization was assessed. In the absence of test compounds and RNA, NP migrated to a position corresponding to low-molecular-weight NP (lane 1). The intensity of the low-molecular-weight NP band decreased as the RNA concentration was increased, and high-molecular-weight bands were observed at the top of the gel (Figure 1C, lanes 2–5). The effects of NUD-1 (lanes 6–9) and naproxen (lanes 10–13) were analyzed by quantifying the intensity of the high- and low-molecular-weight bands in reference to the corresponding lanes in the absence of the compounds (lanes 2–5).The presence of NUD-1 or naproxen significantly decreased (*p* < 0.05) the intensity of the band representing the high-molecular-weight oligomer (Figure 1D), whereas that of the band representing the low-molecular-weight NP increased (Figure 1D). In addition, the formation of the high-molecular-weight band was suppressed in a dose-dependent manner by NUD-1 or naproxen treatment (Figure S2A,B). Oseltamivir does not bind to NP, thus it did not inhibit NP oligomerization (Figure S2A, lane 12). These results indicate that NUD-1 and naproxen interfered with the formation of high-molecular-weight NP oligomers. We also confirmed the reliability of BN-PAGE by control experiments (Figure S2C–F). Analysis in denatured condition by SDS-PAGE showed similar amounts of NP were loaded onto BN-PAGE in all test conditions, although NP easily oligomerized (Figure S2C,D). DMSO concentration up to 4% did not interfere with the formation of the high-molecular-weight NP (Figure S2E). Also, treatment of NP with NUD-1 and naproxen in the absence of RNA did not affect NP migration (Figure S2F). Furthermore, in silico analysis was performed to determine the interaction of NUD-1 with the RNA binding region and tail-binding pocket of NP, the site of NP oligomerization (Figure S3). Molecular docking simulations were performed using UCSF DOCK (version 6.7) [36,37], and the stability of NUD-1 binding to RNA-binding region and tail-binding pocket was assessed by performing molecular dynamics simulations at 310 K (36.85 °C) and 1 atm using Gromacs (version 5.1.4) software [38]. Amber ff99SB-ILDN force field [39] was used for NP, and general amber force field (version 2.1) [40] was used for NUD-1. The compound showed weak interaction with the RNA-binding region, whereas it stably bound to the NP tail-binding pocket, supporting the inhibition of NP oligomerization by NUD-1.

### 3.2. NUD-1 Inhibits Viral Transcription Activity

**Figure 2:**
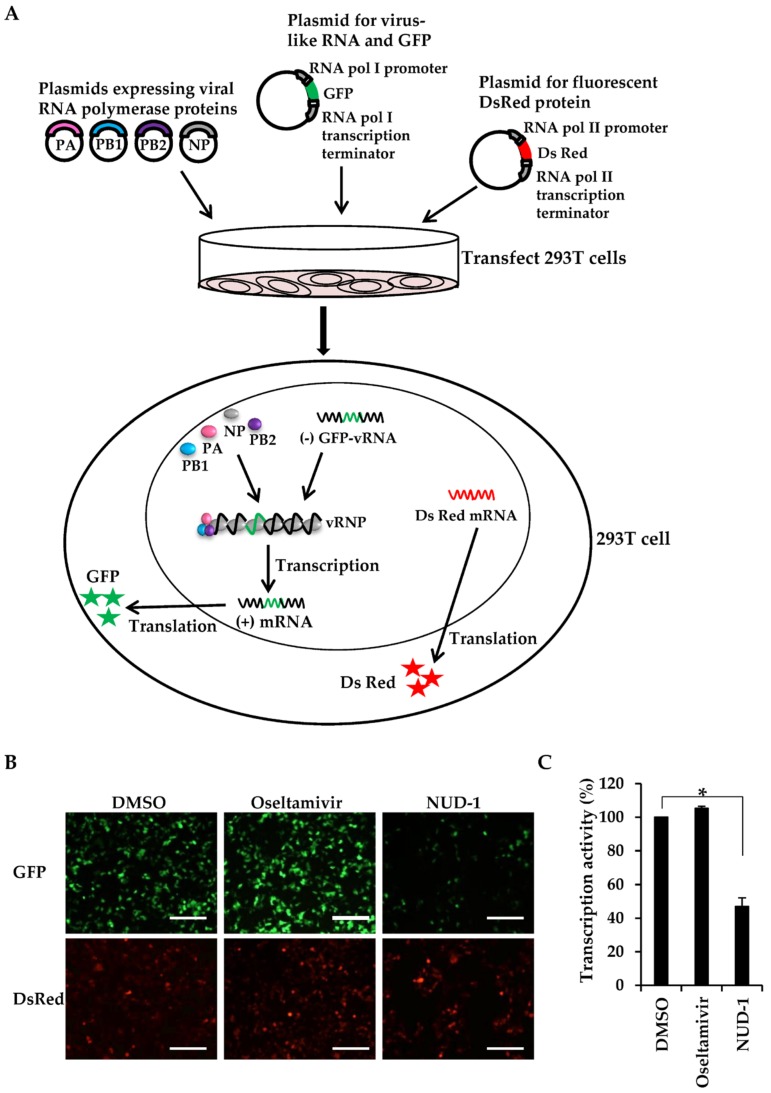
Inhibitory effects of NUD-1 on viral transcription activity. In this experiment, 293T cells were co-transfected with the viral protein expression plasmids (pCAGGS-PA-WSN, pCAGGS-PB1-WSN, pCAGGS-PB2-WSN, and pCAGGS-NP-WSN), the model viral genome expression plasmid (pPolI/NP(0)GFP(0)), and the control plasmid pDsRed2-monomer-N1. Two hours post-transfection, the cells were treated with DMSO, oseltamivir (10 μM), or NUD-1 (10 μM) and further incubated for 24 h. (**A**) Illustration of the miningenome reporter system. (**B**) Transcription inhibition was assessed using a fluorescence microscope. Representative data from two independent experiments are shown. Scale bar, 200 μm. (**C**) Transcription activity (%) was calculated as the ratio of the number of GFP- and DsRed-positive cells in the presence of oseltamivir or NUD-1 in reference to the ratio of GFP- and DsRed-positive cells in DMSO-treated cells. Means ± standard deviations from three different microscopy fields are shown. Statistical analysis was done in comparison to the DMSO control. The asterisk indicates *p* < 0.001.

Because NP oligomerization is important for the formation of the vRNP complex, the transcription template in influenza virus, we investigated whether NUD-1 interferes with transcription activity. We used a minigenome reporter system [28,41], in which vRNPs can be reconstituted by transfecting cells with plasmids expressing the vRNP components (virus-like RNA, PB1, PB2, PA, and NP). The negative strand of the viral RNA genome encoding GFP is transcribed in the cells under the control of cellular RNA polymerase I promoter and forms a complex with polymerase proteins to form vRNP (Figure 2A). Thus, GFP is expressed upon the successful formation of vRNP and RNA-dependent RNA polymerase transcription activity in 293T cells (Figure 2A). DsRed fluorescent protein expression is driven by cellular RNA polymerase II, hence was used as a control. Using this system, we first confirmed that favipiravir, a known viral transcription inhibitor, showed dose-dependent inhibition of GFP expression (Figure S4). Thus, the effect of NUD-1 on viral transcription was tested. The expression of DsRed was similar in DMSO-, oseltamivir-, and NUD-1-treated cells, meaning that NUD-1 did not exhibit cytotoxic effects, which is in accordance with the cell viability assay results in Figure S1C. The cells treated with DMSO and oseltamivir expressed GFP at comparable levels, indicating successful transcription activity (Figure 2B,C). As expected, GFP expression was significantly inhibited in the presence of NUD-1, denoting the suppression of viral transcription activity.

### 3.3. NUD-1 Suppresses the Expression of Late Viral Proteins

**Figure 3:**
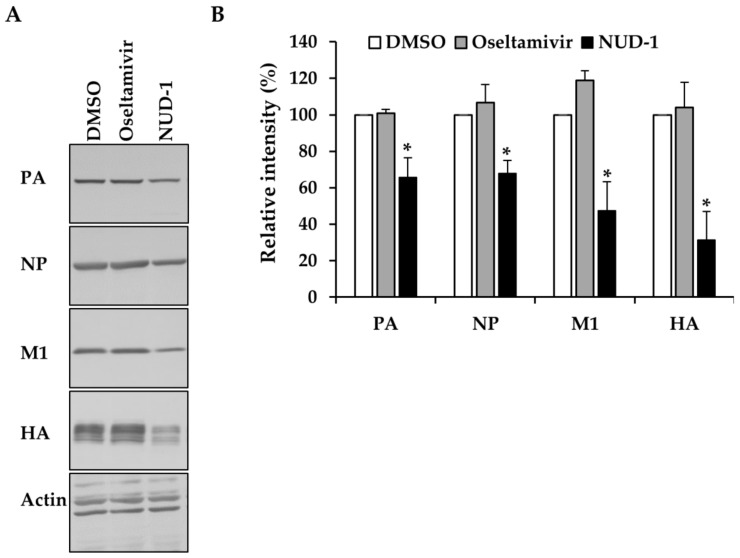
Effect of NUD-1 on the expression of viral proteins. Madin–Darby canine kidney cells were infected with A/WSN/33 virus (multiplicity of infection = 1) in the presence of oseltamivir (100 μM), NUD-1 (12.5 μM), or DMSO. Nine hours post-infection, the cells were collected for Western blotting. (**A**) Viral protein expression was detected by Western blotting using anti-PA, anti-NP, anti-M1, anti-HA, and anti-actin antibodies. (**B**) The band intensity was quantified using ImageJ software. Relative band intensity (%) in the presence of NUD-1 and oseltamivir was calculated in reference to DMSO (control). The level of actin was used for normalization. The data represent the means and standard deviations of three independent experiments. Statistical analysis was performed in comparison to DMSO control. The asterisk indicates *p* < 0.01.

In the course of viral replication, newly synthesized polymerase proteins and NP are imported into the nucleus, where they are assembled into cRNPs and vRNPs to support late transcription [42,43]. We thus investigated the effect of NUD-1 on the expression of early (NP and PA) and late (M1 and HA) viral proteins. The expression of viral proteins in cells treated with oseltamivir or DMSO was not inhibited (Figure 3). Oseltamivir inhibits virus replication by preventing the release of progeny virions from infected cells; hence, it does not interfere with protein synthesis. By contrast, in the presence of NUD-1, viral protein levels were reduced. The extent of inhibition of late viral proteins (M1 and HA) was notably greater than that of early viral proteins (NP and PA), suggesting the impairment of NP oligomerization and thus, reduced formation of cRNP and vRNP for late transcription.

### 3.4. Nuclear Accumulation of NP in NUD-1-Treated Cells

**Figure 4:**
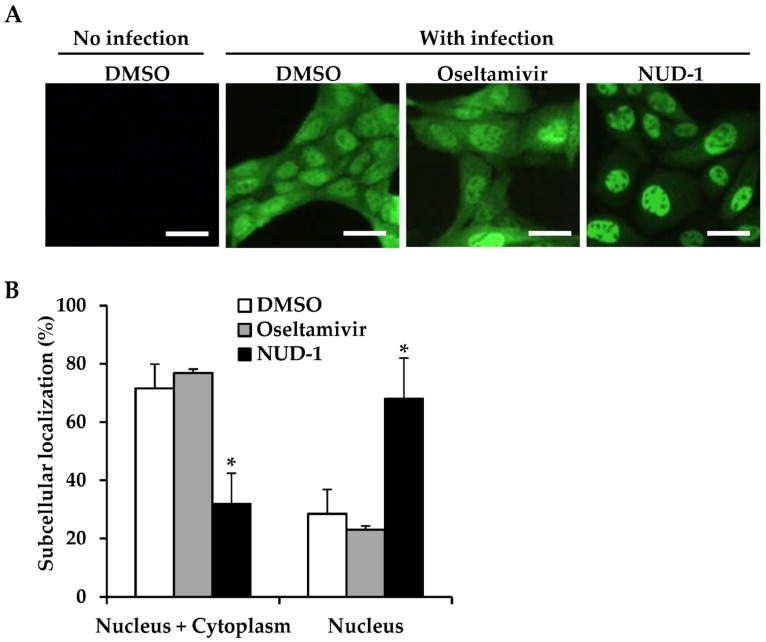
Subcellular localization of nucleoprotein (NP). Madin–Darby canine kidney cells were infected with the A/WSN/33 virus (multiplicity of infection = 5) in the presence of DMSO, oseltamivir (16 μM), or NUD-1 (16 μM) and incubated for 9 h. (**A**) The cells were fixed and stained with anti-NP antibody followed by Alexa Fluor 488-conjugated secondary antibody to determine the subcellular localization of NP. One representative result from three independent experiments is shown. Scale bar, 25 μm. (**B**) The percentage of cells exhibiting both nuclear and cytoplasmic localization and only nuclear localization of NP was calculated by counting two microscopy fields per experiment. The presented data are the mean and standard deviation of three independent experiments with statistical analysis in comparison to DMSO control. The asterisk indicates a *p* value of less than 0.05.

Previous studies found that NP oligomerization is crucial for NP export from the nucleus, as NP mutants that lack the ability to form NP oligomers were found to remain localized in the nucleus [16,44]. Thus, we investigated the effect of NUD-1 on the subcellular localization of NP in infected cells (Figure 4). In oseltamivir-treated cells, 77% of cells displayed an equal distribution of NP in the nucleus and cytoplasm, whereas 23% of cells exhibited nuclear accumulation of NP (Figure 4B), similar what observed in DMSO-treated cells. Conversely, 68% of NUD-1–treated cells exhibited nuclear localization of NP. Similar results were observed 6 and 12 h post-infection (Figure S5). Naproxen-treated cells also exhibited nuclear accumulation of NP (Figure S5). These results support the inhibition of NP oligomerization by NUD-1, leading to NP retention in the nucleus.

### 3.5. Serial Passage of Influenza Virus in the Presence of NUD-1

To determine whether NUD-1 induces the generation of resistant variants, the A/California/7/2009 virus was serially passaged in the presence of increasing concentrations of NUD-1 (Table 1 and Figure S6). The virus passaged in the absence of a drug retained similar sensitivity to oseltamivir acid and NUD-1 as the unpassaged virus. The passage of the virus in the presence of oseltamivir acid resulted in the loss of drug sensitivity after five passages, with more than a 5000-fold increase in IC_50_. The passage of the virus in the presence of NUD-1 did not affect its sensitivity even after nine passages. Notably, the oseltamivir acid-resistant virus was sensitive to NUD-1. Thus, in our experimental conditions, NUD-1 did not induce the generation of drug-resistant mutants and retained efficacy against oseltamivir-resistant viruses.

## 4. Discussion

In our recent work [22], we identified NUD-1 via in silico screening of a chemical library of approximately 10,000 compounds to find compounds that bind to the tail-binding pocket of NP. Surface plasmon resonance assay and fragment molecular orbital calculations showed that NUD-1 could bind to NP and strongly interact with E339, a key amino acid in the formation of NP oligomers. Time-of-addition experiments showed that NUD-1 was potent when added to cells 6 h post-infection and removed 9 h post-infection (6–9 h treatment), a time correlating with the assembly of vRNP and its export from the cells [45,46,47]. In the present study, we investigated the inhibition of NP oligomerization using assays that mimic the process of vRNP formation. The determination of RNP structure using cryoelectron microscopy has enhanced the understanding of how NP forms oligomers and binds RNA in a native vRNP [48,49]. NP has head, body, and tail domains. Between the head and the body domains, there is an arginine-rich groove that binds to the viral RNA, and at the opposite side is a tail-binding pocket that interacts with the tail of adjacent NP monomers to form oligomers. The NP oligomers are stabilized by a salt bridge between E339 present in the tail-binding pocket and R416 in the tail of NP [23,50]. In this study, we treated recombinant NP with RNA and found that the addition of NUD-1 reduced the formation of NP oligomers (Figure 1). Furthermore, the in silico analysis of the interaction between NUD-1 and NP showed stable binding of the compound to the tail-binding pocket, but weak interaction with the RNA-binding region (Figure S3). In a minigenome system, where cells were transfected with plasmids expressing the vRNP complex proteins, the presence of NUD-1 significantly inhibited viral transcription (Figure 2), suggesting the abrogation of vRNP reconstitution. Moreover, in cells infected with the virus and treated with NUD-1, NP was localized in the nucleus (Figure 4). These findings are in accordance with other studies showing that NP mutants that did not form oligomers and vRNP were retained in the nucleus [16,44]. Thus, our data demonstrate that NUD-1 interferes with NP oligomerization.

In clinical studies, resistance to oseltamivir [51] and reduced susceptibility to baloxavir [9] have been reported to occur during treatment. Hence, new antiviral targets for influenza treatments are highly needed to enable the discovery of drugs with reduced potential to induce resistance. NP is a highly conserved multifunctional viral protein playing essential roles in virus replication. In 2010, nucleozin was reported as the first inhibitor that induces the formation of high-order oligomers of NP, preventing its nuclear transport and proper binding to viral RNA [20]. However, nucleozin-resistant mutants were subsequently isolated after the serial passage of IAV with a nucleozin analog. Another group reported that naproxen could inhibit NP–RNA binding [17,52], the nuclear export of NP [53], and serial passage experiments did not generate resistant variants. A compound 3 and its analogs were also revealed to inhibit viral replication by disrupting the conserved E339–R416 salt bridge, which is essential for NP trimerization [18,19]. These studies validate NP as a potential target for drug development. We identified NUD-1 as an inhibitor of NP oligomerization and investigated whether it induced the generation of resistant mutants by serially passaging a clinical isolate of the 2009 H1N1 pandemic influenza virus (A/California/7/2009). The 2009 H1N1 pandemic flu virus is currently circulating as a seasonal H1N1 influenza strain; therefore, we used this strain instead of a laboratory strain to illustrate the potency of NUD-1. In our study, NUD-1 did not select for resistant mutants after nine passages, implying that the NP target site for NUD-1 is stable under drug pressure. The NP tail-binding pocket which is the predicted binding site for NUD-1 is highly conserved [22,54]. The E339 amino acid that showed strong interaction with the compound is 100% conserved in IAV, and mutational analysis studies revealed that recombinant viruses having mutations in E339 could not be rescued in reverse-genetics experiments [50,55]. This suggests that a E339 mutant virus may not exist; therefore, our in vitro serial-passage experiments were unable to generate escape mutants. However, further investigations using in vivo and in silico experiments are needed to determine the genetic barrier to drug resistance for NUD-1.

In conclusion, NUD-1 displays a novel mechanism of action and low possibility of inducing the generation of resistant mutants. Numerous 4-hydroxyquinolinone derivatives are currently in clinical use such as roquinimex [56], decoquinate [57], laquinimod [58], paquinimod [59], and tasquinomod [60]. Thus, NUD-1 can serve as a basis for novel anti-influenza drug development through structural modifications.

## Figures and Tables

**Table 1:** Sensitivity of the passaged A/California/7/2009 virus to oseltamivir acid and NUD-1.

	50% Inhibitory Concentration (µM)	
	Oseltamivir acid	NUD-1
Unpassaged virus	0.02 ± 0.00	2.23 ± 0.04
Passage 9 in the absence of drug	0.01 ± 0.00	1.90 ± 0.28
Passage 5 in the presence of oseltamivir acid	>100	1.75 ± 0.07
Passage 9 in the presence of NUD-1	0.02 ± 0.00	1.92 ± 0.01

## Supplementary Materials

The following are available online at https://www.mdpi.com/1999-4915/12/3/337/s1: Figure S1. Chemical structure, antiviral activity and cytotoxicity of NUD-1; Figure S2. Effect of NUD-1 and naproxen on NP oligomerization; Figure S3. In silico analysis of the interaction between NUD-1 and nucleoprotein (NP); Figure S4. Viral transcription assay; Figure S5. Subcellular localization of nucleoprotein (NP) in NUD-1 and naproxen-treated cells; and Figure S6. Serial passage of influenza A virus in the presence of oseltamivir acid and NUD-1.
