# Supplementary material for: A Quinolinone Compound Inhibiting the Oligomerization of Nucleoprotein of Influenza A Virus Prevents the Selection of Escape Mutants
Source: Viruses. 2020 Mar 19;12(3):337. doi: 10.3390/v12030337 (PMC7150793; doi:10.3390/v12030337)
Supplement: Supplementary file 1 [file viruses-12-00337-s001.pdf]

Supplementary Material

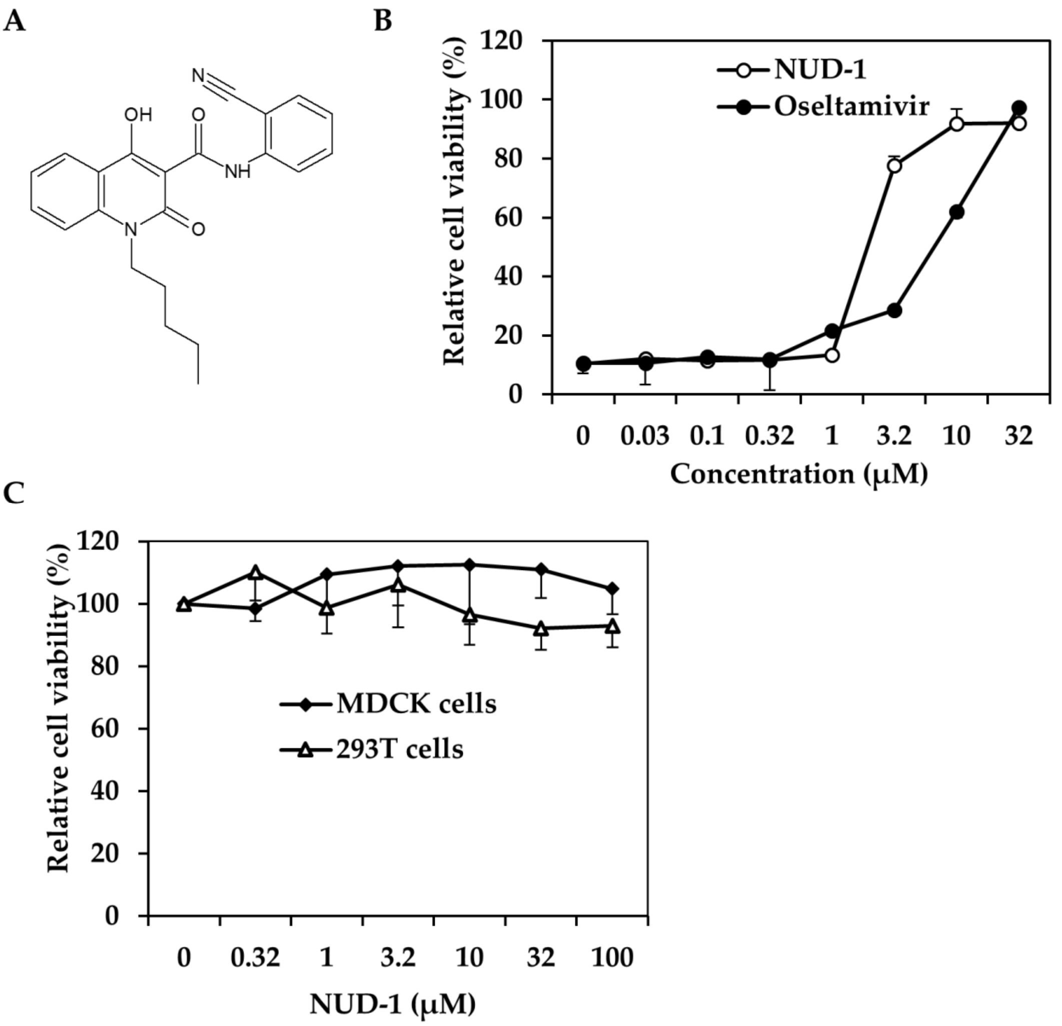

Figure S1. Chemical structure, antiviral activity, and cytotoxicity of NUD-1. (**A**) NUD-1 (N-(2-cyanophenyl)-1,2-dihydro-4hydroxy-2-oxo-1-pentyl-3-quinolinecarboxamide, molecular weight = 375.4). (**B**) The antiviral activity of NUD-1 and oseltamivir phosphate was determined using the crystal violet assay. Madin–Darby canine kidney (MDCK) cells were seeded in 96-well tissue culture plates and incubated for 24 h at 37 °C. NUD-1 and oseltamivir were serially diluted with minimum essential medium (MEM) containing 1% 100× MEM vitamin solution (MEM vitamin). The cells were washed with FBS-free MEM, and 100 μL of serially diluted compounds were added to the cells, followed by the addition of 100 μL of virus solution (1000 $TCID_{50}$/mL of A/WSN/33 in MEM vitamin). The cells were incubated at 37 °C for 48 h before fixing with 70% EtOH and staining with 0.5% crystal violet. The plates were washed and air-dried at room temperature, and the optical density at 560 nm was measured using an Infinite M200 plate reader. The relative cell viability (%) in wells treated with compounds was calculated in reference to the uninfected untreated control.

The mean and standard deviation from three independent experiments is shown. NUD-1 exhibited potent antiviral activity at submicromolar concentrations. (**C**) The cytotoxicity of NUD-1 was determined using the water-soluble tetrazolium salt (WST-1) assay. MDCK cells and 293T cells were seeded in 96-well tissue culture plates at a density of 3×10^4 cells/well and incubated for 24 h. The medium was then replaced with 100 μL of MEM vitamin containing varying concentrations of NUD-1. Dulbecco's minimum essential medium was used for 293T cells culture. After incubating MDCK cells for 48 h and 293T cells for 24 h, 10 μL of Cell Proliferation Reagent WST-1 (Roche, Germany) was added to the cells, and incubation was continued at 37°C for 30 min. Absorbance was measured at 450 and 600 nm as the reference wavelength, using a plate reader. The relative cell viability in treated cells was calculated in reference to untreated cells.

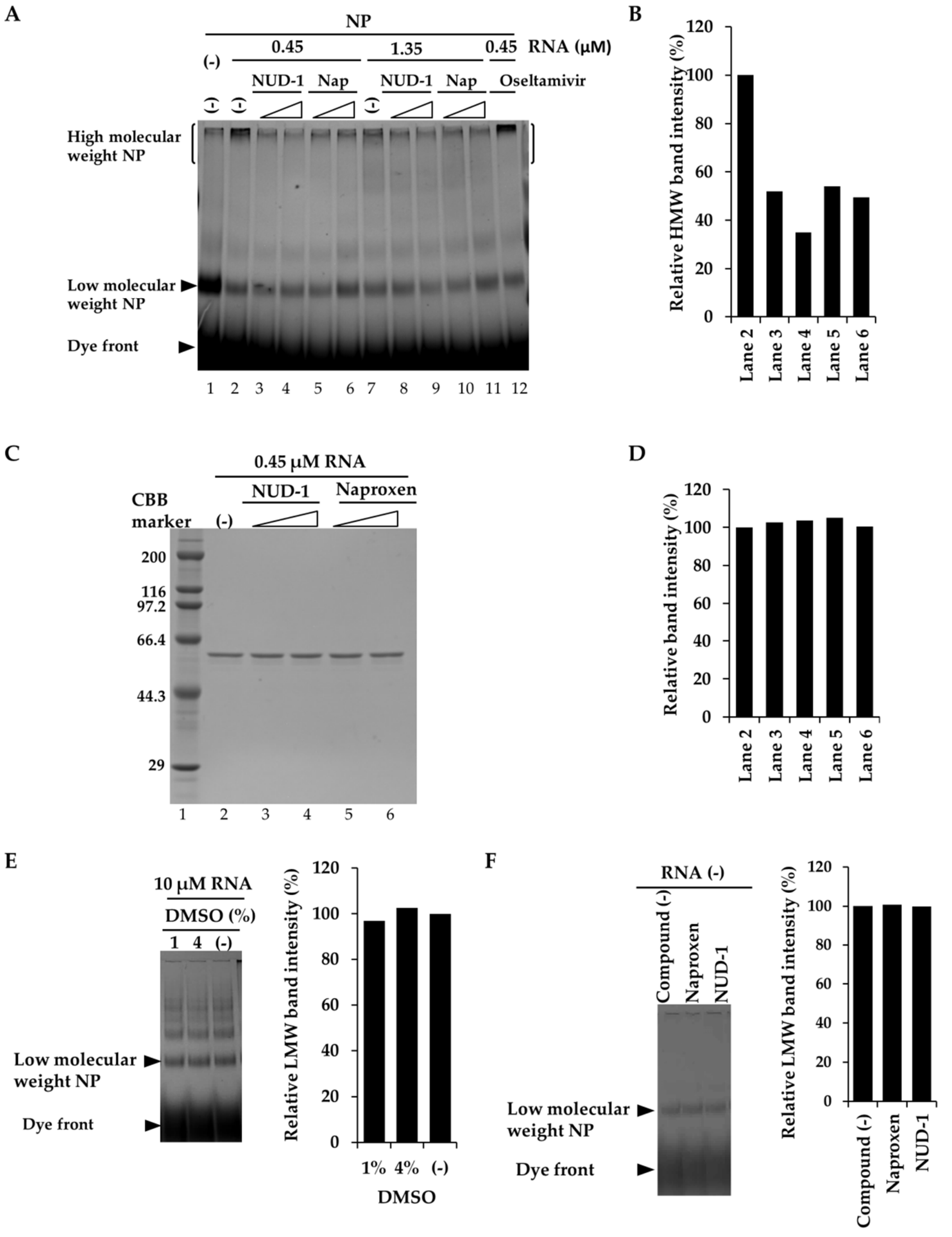

Figure S2. Effect of NUD-1 and naproxen on NP oligomerization. (**A**) To analyze the dose-dependent inhibition of NP oligomerization by NUD-1 and naproxen, NP (2.5 μM, equivalent to 2 μg) was mixed with RNA (0.45 or 1.35 μM) in the absence of any compound (lanes 2 and 7) or in

the presence of 100 μM (lanes 3, 5, 8, and 10) or 300 μM (lanes 4, 6, 9, and 11) of NUD-1 or naproxen (Nap), or 300 μM oseltamivir (lane 12) and incubated at room temperature overnight before analysis via BN-PAGE. After destaining and thorough washing, the gel was silver-stained n (Cosmo Bio Co. Ltd., Tokyo, Japan) according to the manufacturer's instructions. The intensity of the smear at the top of the gel (enclosed by bracket) was quantified using ImageJ software. The relative band intensity in the presence of NUD-1 or naproxen was calculated in reference to that in the absence of the compounds. Two independent experiments were performed, and representative data are shown. (**B**) The mean band intensity of two independent experiments in panel A was quantified using ImageJ software. Relative band intensities of NP treated with 0.45 μM RNA (no compound, lane 2; 100 μM NUD-1, lane 3; 300 μM NUD-1, lane 4; 100 μM naproxen, lane 5; 300 μM naproxen, lane 6) are shown. (**C**) The NP samples treated with 0.45 μM RNA in panel A (lanes 2-6) were also analyzed in denatured conditions using 10% SDS-PAGE. (**D**) Band intensities of panel C were quantified using ImageJ software. (**E**) The effect of DMSO on NP oligomerization was analyzed. NP was mixed with 10 μM of RNA in the absence or presence of DMSO and analyzed by BN PAGE. The intensity of low-molecular-weight (LMW) NP was quantified by ImageJ. (**F**) NP was mixed with 100 μM of naproxen or NUD-1 and incubated at RT overnight before analysis by BN-PAGE; the low-molecular weight NP was quantified.

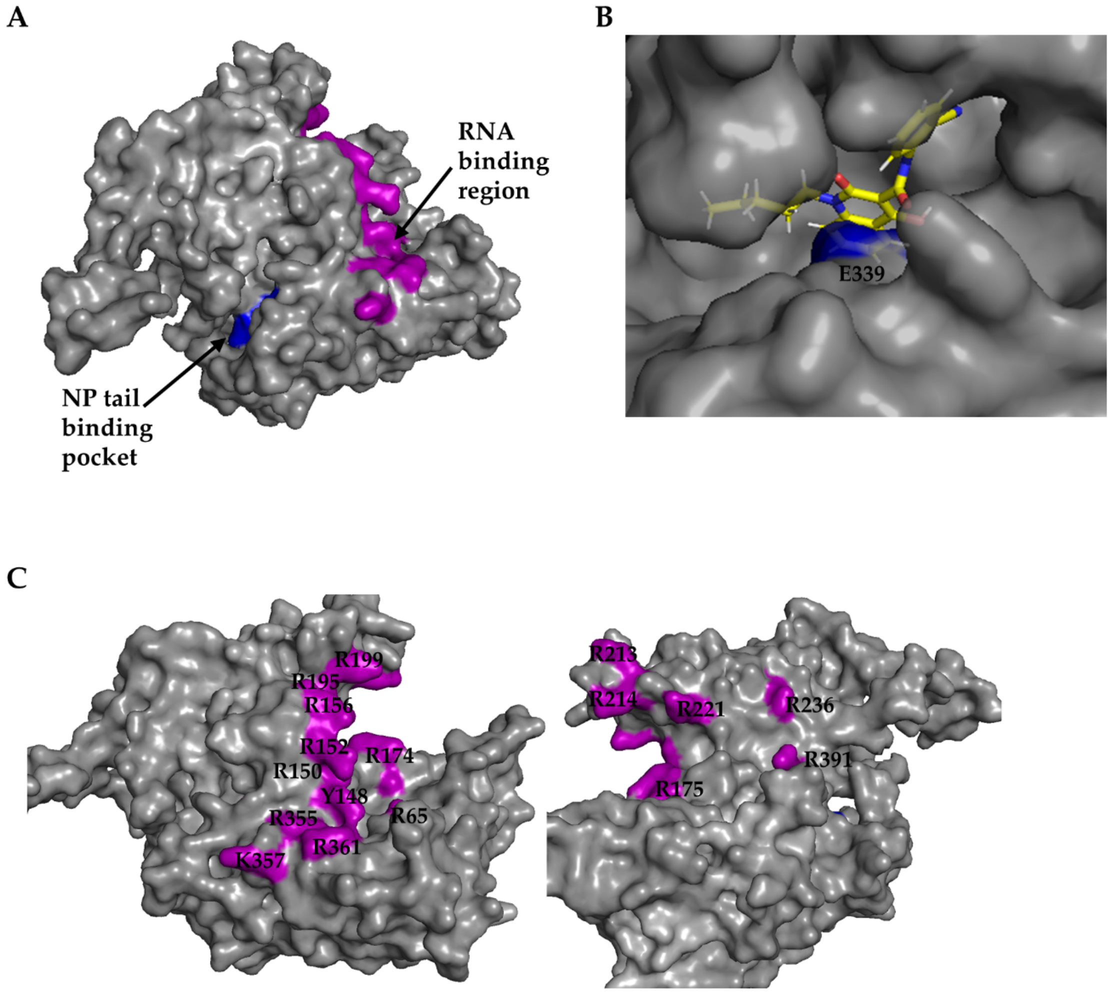

A
RNA binding region
NP tail binding pocket
B
E339
C
R199
R195
R156
R152
R174
R150
Y148
R355
R65
R361
K357
R213
R214
R221
R236
R391
R175

D

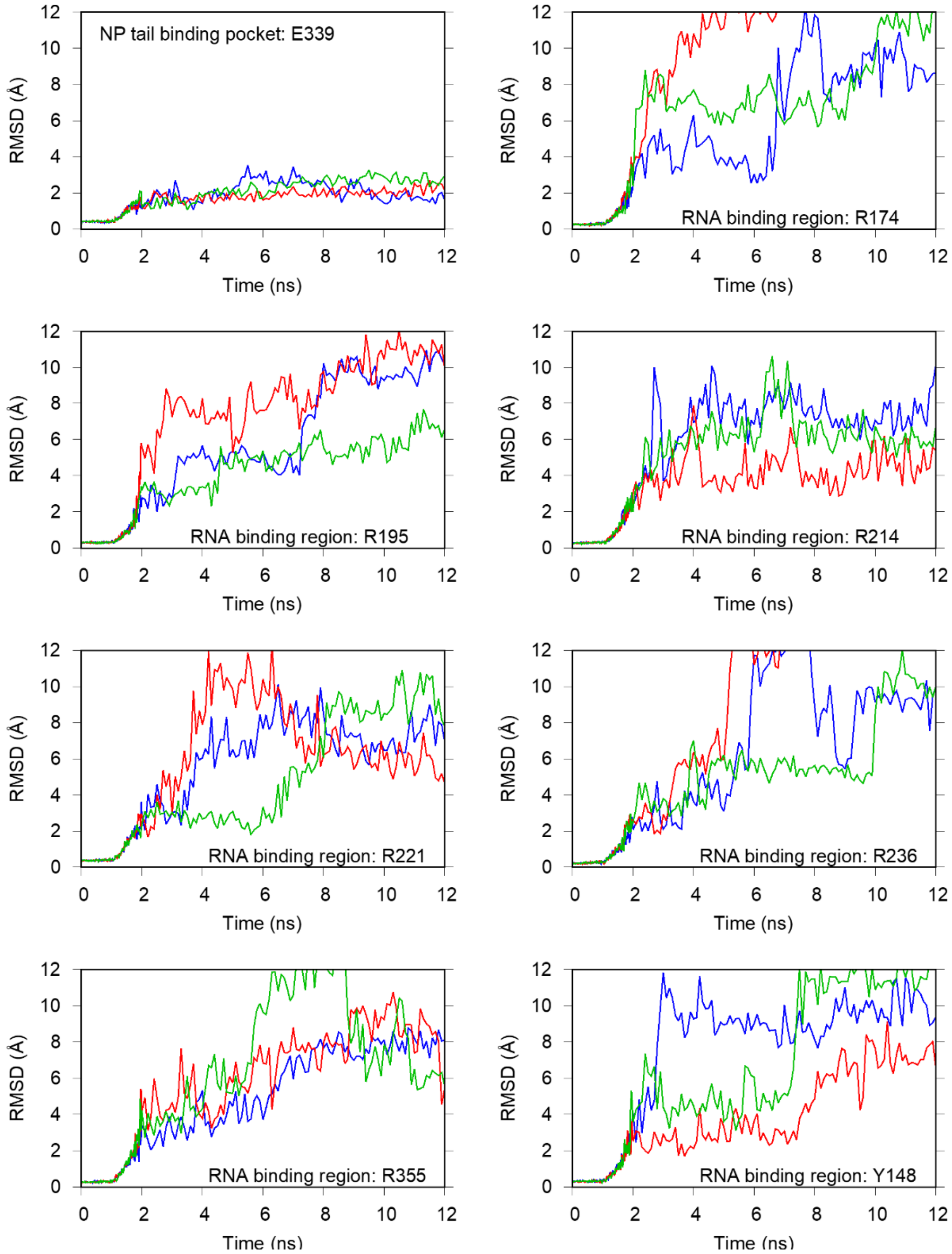

Figure S3. In silico analysis of the interaction between NUD-1 and nucleoprotein (NP). The crystal structure of influenza A virus NP derived from the Protein Data Bank (PDB ID: 2IQH) was used for the in silico analyses. Molecular docking simulations were performed using UCSF DOCK (version 6.7) [1,2] to determine the interactions of NUD-1 with the tail-binding pocket and RNA-

binding region of NP. (**A**) Structure of NP denoting the tail-binding pocket (blue) and RNA-binding region (purple). (**B**) Close view of the molecular docking of NUD-1 to the NP tail-binding pocket. (**C**) The amino acids in the RNA binding groove that interact with viral RNA are shown. For molecular docking of NUD-1 to the RNA binding region, seven docking regions were set, centering on R174, R195, R214, R221, R236, R355, and Y148. (**D**) The stability of NUD-1 binding poses generated with docking experiments were assessed by performing molecular dynamics (MD) simulations at 310 K and 1 atm using Gromacs (version 5.1.4) software [3]. Amber ff99SB-ILDN force field [4] was used for NP and general amber force field (GAFF; version 2.1) [5] was used for NUD-1. Three independent MD runs were performed for each binding pose for 2 ns of equilibration run with releasing restraints and 10 ns of production run with the time step of 2 fs. Heavy-atom root-mean-square deviation (RMSD) of NUD-1 with respect to binding pose obtained from the docking simulation was calculated; RMSDs for MD runs 1, 2, and 3 are shown in blue, red, and green lines, respectively. A small RMSD means that the NUD-1 has stable binding to NP, whereas a large RMSD denotes unstable binding. The results illustrate that NUD-1 binds strongly to the NP tail-binding pocket but weakly interacts with the RNA-binding region.

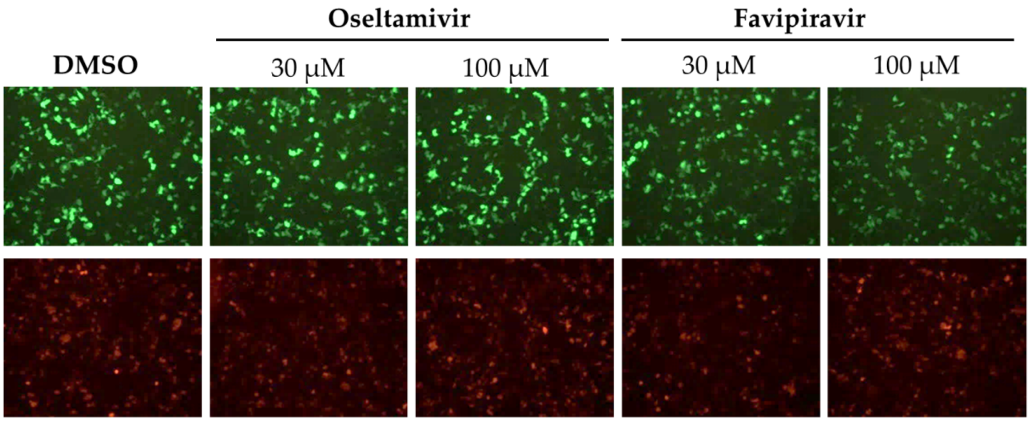

Figure S4. Viral transcription assay. In this experiment, 293T cells seeded into 24-well plates were transfected with plasmids expressing the vRNP complex proteins (pCAGGS-PA-WSN, pCAGGS-PB1-WSN, pCAGGS-PB2-WSN, and pCAGGS-NP-WSN), the model viral gene expression plasmid pPolI/NP(0)GFP(0), and pDsRed2-monomer-N1. Two hours post-transfection, the medium was replaced with 500 μL of DMEM containing serially diluted compounds and 25 mM HEPES (pH 7.4), and incubation was protracted for 24 h. The expression of GFP and DsRed proteins was observed via fluorescence microscopy. The presence of DMSO and oseltamivir did not inhibit the expression of GFP, while favipiravir suppressed GFP expression in a dose-dependent manner.

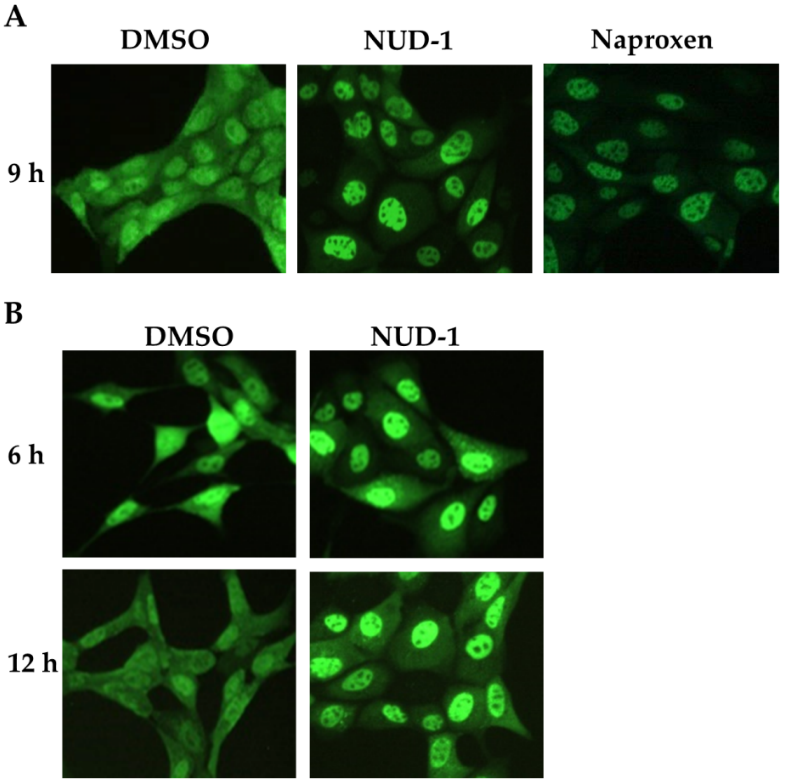

Figure S5. Subcellular localization of nucleoprotein (NP) in NUD-1- and naproxen-treated cells. Madin–Darby canine kidney cells were infected with the A/WSN/33 influenza virus strain (multiplicity of infection = 5) in the presence of DMSO, 16 μM of NUD-1, or naproxen and incubated at 37 °C. (**A**) Nine hours post-infection, the cells were fixed and stained with an anti-NP antibody followed by Alexa Fluor 488-conjugated secondary antibody. DMSO-treated cells exhibited both nuclear and cytoplasmic localization of NP, whereas NUD-1- and naproxen-treated cells displayed nuclear localization of NP. **(B**) To check NP localization in NUD-1-treated cells at different time points during the virus replication cycle, the cells were fixed and stained 6 and 12 h post-infection. Similarly, NP was retained in the nucleus.

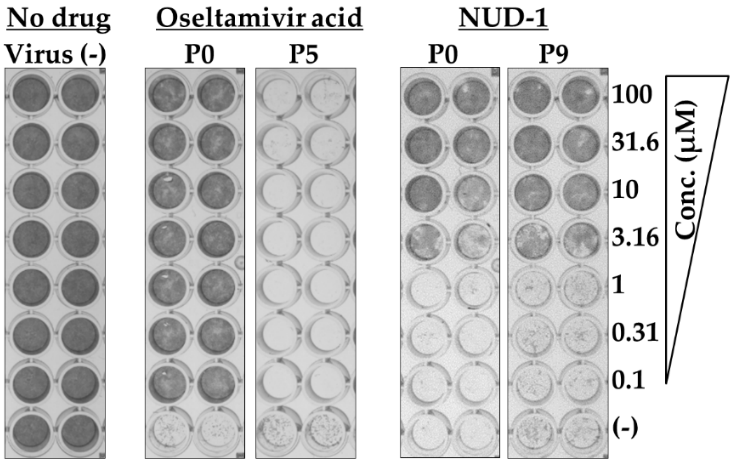

Figure S6. Serial passage of influenza A virus in the presence of oseltamivir acid and NUD-1. A clinical isolate of the 2009 pandemic influenza virus strain (A/California/7/2009) was serially passaged under drug pressure. The virus was grown in the presence of increasing concentrations of either oseltamivir acid or NUD-1. In the first passage, Madin–Darby canine kidney cells were infected at a multiplicity of infection of 0.001 in the presence of oseltamivir acid (0.001–0.1 μM) or NUD-1 (1–8 μM). These concentrations were selected on the basis of the 50% inhibitory concentrations of oseltamivir acid (0.02 μM) and NUD-1 (2.2 μM). The cells were harvested after 3–4 days of incubation when a cytopathic effect was evident, and the viral titers were determined using the hemagglutination assay. After identifying the highest concentration of oseltamivir acid or NUD-1 that permitted detectable viral growth, the supernatant from treated cells was diluted 1000-fold and used to infect new cells. The concentrations of oseltamivir acid and NUD-1 were increased by 10- and 2-fold, respectively, in subsequent passages. The 10-fold increase in the oseltamivir acid concentration was associated with a rapid increase in viral titers in all tested concentrations. After the fifth and ninth passages of the virus in the presence of oseltamivir acid or NUD-1, the culture supernatant was harvested. Viral titers were determined using the $TCID_{50}$ assay, and the sensitivity of the passaged virus to the two drugs was determined using the crystal violet assay. Plaque assay isolation of a resistant clone in the presence of 100 μM oseltamivir was performed after the fifth passage. The sensitivity of unpassaged virus (P0), of the plaque assay isolate of a resistant clone after the fifth passage in the presence of oseltamivir acid (P5), and of the culture supernatant after nine passages in the presence of NUD-1 (P9) to the two drugs was tested in duplicate using the crystal violet assay. The virus passaged in the presence of oseltamivir had developed drug resistance, whereas the virus passaged in the presence of NUD-1 retained similar drug sensitivity as the unpassaged virus.

References

1. Allen, W.J.; Balius, T.E.; Mukherjee, S.; Brozell, S.R.; Moustakas, D.T.; Lang, P.T.; Case, D.A.; Kuntz, I.D.; Rizzo, R.C. DOCK 6: Impact of new features and current docking performance. *J. Comput. Chem.* **2015**, *36*, 1132–1156.
2. Lang, P.T.; Brozell, S.R.; Mukherjee, S.; Pettersen, E.F.; Meng, E.C.; Thomas, V.; Rizzo, R.C.; Case, D.A.; James, T.L.; Kuntz, I.D. DOCK 6: combining techniques to model RNA-small molecule complexes. *RNA N. Y. N* **2009**, *15*, 1219–1230.
3. Abraham, M.J.; Murtola, T.; Schulz, R.; Páll, S.; Smith, J.C.; Hess, B.; Lindahl, E. GROMACS: High performance molecular simulations through multi-level parallelism from laptops to supercomputers. *SoftwareX* **2015**, *1–2*, 19–25.
4. Lindorff-Larsen, K.; Piana, S.; Palmo, K.; Maragakis, P.; Klepeis, J.L.; Dror, R.O.; Shaw, D.E. Improved side-chain torsion potentials for the Amber ff99SB protein force field. *Proteins* **2010**, *78*, 1950–1958.
5. Wang, J.; Wolf, R.M.; Caldwell, J.W.; Kollman, P.A.; Case, D.A. Development and testing of a general amber force field. *J. Comput. Chem.* **2004**, *25*, 1157–1174.
